# Detection of chimeric alpha-defensin transcripts and peptides in mouse Paneth cells

**DOI:** 10.3389/fimmu.2025.1543059

**Published:** 2025-02-06

**Authors:** Steven Timmermans, Charlotte Wallaeys, Somara De Beul, Natalia Garcia-Gonzales, Claude Libert

**Affiliations:** ^1^ Center for Inflammation Research, Vlaams Instituut voor Biotechnologie (VIB), Ghent, Belgium; ^2^ Department of Biomedical Molecular Biology, Ghent University, Ghent, Belgium

**Keywords:** alpha-defensin, chimeric protein, *Mus musculus*, Paneth cell, anti-microbial activities

## Abstract

**Introduction:**

In mammals, Paneth cells, located in the crypts of the small intestine, produceantimicrobial peptides that serve to keep the intestinal microbiome under control. a-Defensins are the primary antimicrobial peptides produced by these cells.

**Methods:**

We used 148 publicly available bulk RNA-seq samples on purified PCs, proteomics on enriched purified PC proteins and *Defa* peptide activity assays to detect all *Defa* transcrips, including potential chimeric transcrips.

**Results:**

We identified 28 expressed *Defa* genes in mice, with up to 85% of Paneth cell RNA reads mapping to these genes. Chimeric mRNAs, involving sequences from two different *Defa* genes, were detected in most experiments. Despite their low abundance (less than 0.3%), mass spectrometry confirmed the presence of chimeric peptides. Synthetic versions of these peptides demonstrated antibacterial activity against multiple bacterial species.

**Conclusion:**

We show the existence of chimeric *Defa* transcripts and peptides in mice that are biologically active. We propose a possible stochatic mechanism or that the activation of the UPR patway may play a role in their production

## Introduction

Paneth cells (PCs) are secretory epithelial cells found in the small intestinal crypts of Lieberkühn in mammals (1, 2). They play a crucial role in regulating the composition and containment of the enteric microbiota and thus in maintaining gut homeostasis, by secreting antimicrobial peptides (AMPs) (3), such as lysozyme-1 (4) and α-defensins (5). Paneth cells also help in keeping the neighboring stem cells undifferentiated by Wnt signaling and secretion of metabolites (6).

Mature, secreted α-defensins contain six conserved cysteines that form three intramolecular disulfide bonds, which stabilize a β-sheet structure which is important for biological activity (7). The antimicrobial activity of α-defensins is dictated by their cationic amino acids and the amphiphilic protein structure, which cause electrostatic interactions with phospholipids of bacterial membranes. This leads to pore formation in the membrane in Gram-positive and Gram-negative bacteria, causing their death. Moreover, α-defensins sequester Lipid II, a precursor molecule essential for bacterial cell wall synthesis (7).

α-defensins are initially synthetized as pre-pro-peptides of about 92 amino acids (AA) long. They lose their 19 AA signal peptide (becoming pro-peptides of about 73 AA long) in the Golgi complex after syntheses and before transport to secretory vesicles, where proteolytic cleavage converts them from pro-defensins to active α-defensins, usually around 33 AA in size (8, 9). In human PCs, this process is performed by trypsin, which is stored as a proenzyme (trypsinogen) that is activated during or shortly after secretion (10). In mouse PCs, the cleavage is performed by matrix metalloproteinase 7 (MMP7), also known as matrilysin (8). The importance of α-defensins in microbial control was demonstrated by several authors. MMP7-deficient mice suffer from lack of α-defensin maturation and hence have no control on their microbial communities and are also super-sensitive to infections (9). Equally so, the cytokine tumor necrosis factor (TNF) was recently shown to undermine AMP production of Paneth cells, leading to uncontrolled bacterial spreading from gut lumen to organs (11). Paneth cells can be sorted by FACS and single cell RNA-seq revealed that such sorts lead to 99.9% pure Paneth cells, which can be studied by bulk RNA-seq or mass spectrometry (12).

The extensive α-defensin (*Defa*) locus on mouse chromosome 8 (0.71 MB in size, viz. chr8:21,515,561-22,225,487) has a complex and dynamic annotation history that is quite confusing. The three primary sources of annotation information in mice are the following: (i) RefSeq, a database of curated genes and transcripts, published and maintained by the National Center for Biotechnology Information (NCBI) (13), (ii) GENCODE, which is part of the Encode project and consists of the manually curated Ensembl-Havana annotation as well as the computational Ensembl-genebuild automated gene annotation (14) and (iii) mouse genome informatics (MGI), an Ensembl database maintained by The Jackson Laboratory (15).

Our interest in Paneth cells has induced us to study and update the actual structure of the *Defa* locus. Purification of Paneth cells followed by bulk RNA-seq has led to the discovery of many mRNA species that contain sequences of multiple (usually 2) different *Defa* genes. These chimeric mRNAs are quite abundant and at least some appear to be recovered as peptides via mass spectrometry. *In vitro* synthetized and tested chimeric peptides contain antimicrobial activity. The mechanism and necessity of their formation remain enigmatic.

## Materials and methods

### Statement regarding sex as a biological variable

Our study uses GEO datasets which contain both male and female animals and sex is not considered as a relevant biological variable in this context.

### Mice

C57BL/6J mice were housed in a specific pathogen free (SPF) facility in individually ventilated cages. They were kept under a 14/10-hour light/dark cycle and were provided access to food and water *ad libitum.*

The mutant mice, expressing a Defa30 protein with C-terminal His6 tag were generated as follows (see **Supplementary Figure 2**). The His_6_ tag along with a glycine-serine-glycine (GSG) linker were inserted right before the *Defa30* (ENSMUSG00000074444) stop codon of C57BL/6J mice using Crispr-Cas9 by the Transgenic Core Facility (TCF) of the Inflammation Research Center (IRC) of VIB-UGent. The Cas9 RNP complex along with guide RNA sequence.

5’ CATGGTCATCTTGTTCTCTG 3’ and single stranded DNA oligo with sequence 5’AGAAGTGGTCATCAGGCACCAGCGTCAGTGGCCTCAGTACTCATGGTCATCTTGTTCTCTcTGGTCTCCATGTTCAgtggtgatggtgatgatgtccggagccGCGACAGCAGAGCATGTACATTAAATGACCCTTACTGCAGGTCCCATTCATGCGTTCTCT3’ were electroporated in C57BL/6J zygotes, followed by transfer to pseudopregnant mice. After birth, mice were screened for the mutation. Several founders were identified and crossed with C57BL/6J and transgenic families propagated. Transgenic animals were used as heterozygotes (Defa30His6^Tg/+^) for Paneth cells sorting and proteomic analysis. All base annotations are according to C57BL/6J genome assembly GRCm39.

### Non-mouse α-defensin prediction

Non-mouse α-defensin annotations were obtained from the UCSC genome browser and placed in a tab delimited text file for processing. Firstly, a coarse gene size-based filtering was applied to the predicted genes, where genes < 500bp or > 3000bp were excluded. Retained genes were then manually further processed: the gene structure was inspected, where possible, for the typical α-defensin 2 exon structure, sequence homology with mouse α-defensin and the fact that a mature peptide was coded for. In addition, any overlapping regions that showed at least 75% overlap were collapsed into one region.

### The construction of a set of theoretical alpha defensins

In order to create a set of potential chimeric alpha defensin transcripts, all 28 existing *Defa* genes were used in a pairwise combination for creating exon 2-based chimers, with each gene acting as donor and acceptor. Each parental pair was used to derive multiple possible chimeric transcripts, based on the following parameters: (i) the resulting chimer is in-frame and hence, transcripts that resulted in a frameshift causing an early stop codon, or loss of the typical cysteine structure were discarded. (ii) The chimer was different from the parentals, at both the transcript and the protein level and (iii) a sequence was discarded if it was the same as one already present in the set from the same donor and acceptor parent. The theoretical chimers from each donor-acceptor pair were merged. Chimers were *in silico* translated and any that were identical to any one of the parental defensins at the protein level were removed from the dataset. Finally, all chimers in the set were compared to each other and those with identical nucleotide sequences were collapsed into one sequence. This resulted in a final set of 2447 sequences that could theoretically be formed from all donor-acceptor combinations.

### PC purification and bulk RNA-seq

Over the past years (2021-2024), our research team has sorted Paneth cells from C57BL/6J mice, as described in (12) and as shown in **Supplementary Figure 1**. Sorted Paneth cells proved to contain less than 0.1% contaminating cells. From 148 mice, we have obtained successfully about 25,000 Paneth cells, and bulk RNA-seq data. For the current study, we have used these RNA-seq datasets, which have been published previously and have the following GEO identification codes (**Table 1**):

**Table 1:** Overview of the GEO datasets used in this work.

GEO id	Description
GSE237588	Gene expression by RNA-seq in purified Paneth cells in antibiotics microbiome depleted mice
GSE237759	Gene expression by RNA-seq in purified Paneth cells of WT and Paneth cell specific P55 KO mice 3 hours after TNF
GSE255507	RNA-seq on FACS sorted Paneth cells from the murine duodenum, jejunum and ileum
GSE267790	Paneth cell TNF-signaling induces bacterial sepsis: PC transcriptome 15h after TNF
GSE267927	Paneth cell TNF-signaling induces bacterial sepsis: PC transcriptome 15h after TNF in IFNARKO mice
GSE269510	Transcriptome of purified PC and non-PC fractions: GR-WT & GR-KO
GSE281682	Paneth cell gene expression with and without zinc in different microbiome contexts (antibiotics, germ-free, normal)
GSE281683	Effect of TNF of gene expression in mouse Paneth cells after 3 hours
GSE281798	Paneth cell gene expression profiles after zinc supplementation
GSE281799	Paneth cell gene expression profiles after zinc supplementation in TNF context
GSE281800	Expression profiling of purified cells after (mature) Paneth cell depletion
GSE281801	The effect of Zinc deficiency on the mouse Paneth cell transcriptome
GSE281875	The effect of Zinc deficiency on the mouse Paneth cell transcriptome in TNF context
GSE281876	Paneth cell transcriptome: effect of DEX on TNF induced inflammatory response

### STAR

A STAR index was created using the GENCODE M30 structural genome annotation GTF and the GRCm39 (mm39) mouse reference genome (using STAR–runMode *genomeGenerate*). All reads were aligned to this index using the default setting of STAR 2.7.10b (16) with the output stored as a bam file (–outSAMtype BAM SortedByCoordinate –outBAMcompression 10 –outBAMsortingThreadN 3 –limitBAMsortRAM 6500000000).

### Salmon

Read quantification was performed with Salmon (17) in read mode, which allows direct quantification from reads. A total of 4 Salmon indices were created for quantification as described in the Salmon manual [Preparing transcriptome indices (mapping-based mode)]: (i) an index of the updated GENCODE transcriptome, (ii) an index of the adapted GENCODE transcriptome with the theoretical chimeric transcripts added (ii) an index of the adapted GENCODE transcriptome with the theoretical chimeric transcripts added as decoy transcripts and (iv) an index of the GENCODE transcriptome with the chimeric transcripts added but with the genomic α-defensin moved to decoy set. Reads were quantified per sample using Salmon quant (options: -l A–validateMappings). The quantifications that were made per sample against each index, expressed as Tags Per Million (TPM) were joined using Salmon quantmerge to 4 tab separated matrix files (one per index).

### Proteomics

#### Sample preparation

Small intestines were excised, rinsed in ice-cold PBS, and cut into small (2–5 mm) pieces. Tissue fragments were incubated in 3 mL lysis buffer (Complete-free EDTA: 1 tablet; DNase (50 mg/ml); 50 mM sodium phosphate, 300 mM NaCl, 10 mM imidazole, pH 7.4) for 20 minutes at 4°C, followed by sonication on ice for 10 minutes to complete lysis. Lysates were centrifugated at 50000 g for 30 minutes. Samples were equilibrated twice with binding buffer Ni Seph (50 mM NaH2PO4, 300 mM NaCl, 10 mM IZ, pH 7.4) with intermediate centrifugation at 5,000 × g for 1 minute to a final volume of 10ml. The sample was then mixed with 10 mL binding buffer and Ni Seph beads and incubated on a stirring wheel at 4°C for 1 hour. Beads were pelleted by centrifugation for 15 minutes at 4347 g, at 4°C and the supernatants were removed. The Ni Seph beads were washed with 10 ml binding buffer Ni Seph. Samples were centrifuged and washed with 5 ml washing buffer Ni Seph (50 mM NaH2PO4, 300 mM NaCl, 20 mM IZ, pH 7.4). Samples were centrifuged and the protein was eluded from the beads with 250 µl elution buffer Ni Seph (50 mM NaH2PO4, 300 mM NaCl, 500 mM IZ, pH 7.4). Concentration was determined with Trinean Dropsense.

For each sample (in 20 mM NaH2PO4 + 500 mM NaCl + 500 mM imidazole pH 7.4) 30 µg protein was taken and ammonium bicarbonate buffer pH8 (ABC) was added to a final concentration of 50mM in 150 µl. Proteins were reduced and alkylated by addition of 10mM TCEP and 40 mM chloroacetamide and incubation for 5 minutes at 45°C in the dark at 750 rpm, followed by a short cooling step on ice. Three aliquots of 10 µg were taken from each sample to perform three separate in-solution digests in parallel by addition of 0.1 µg trypsin (Promega, V5117), 0.1 µg endoproteinase Glu-C (Promega, V1651) or 0.1 µg Elastase (Promega, V1891) to the aliquots. Digestions were incubated for 2 hours at 37°C. The sample pH was lowered to 3 to stop the digestion by addition of trifluoroacetic acid (TFA) to a final concentration of 1%, followed by sample clean-up on a reversed phase (RP) C18 OMIX tip (Agilent). Each tip was first washed 3 times with 100 µl pre-wash buffer [0.1% TFA in water/ACN (20:80, v/v)] and pre-equilibrated 5 times with 100 µl of wash buffer (0.1% TFA in water) before the sample was loaded on the tip. After peptide binding, the tip was washed 3 times with 100 µl of wash buffer and peptides were eluted twice with 100 µl elution buffer [0.1% TFA in water/ACN (40:60, v/v)]. The 2 elutions of each sample were combined in an HPLC vial and dried in a vacuum concentrator.

#### LC−MS/MS analysis

Peptides were re-dissolved in 20µl loading solvent A [0.1% TFA in water/ACN (98:2, v/v)] of which 4µl was injected for LC-MS/MS analysis on an Ultimate 3000 RSLC nano LC (Thermo Fisher Scientific, Bremen, Germany) in-line connected to a Q Exactive mass spectrometer (Thermo Fisher Scientific). a trapping column made in-house, 100 μm internal diameter (I.D.) × 20 mm, 5 μm beads C18 Reprosil-HD, Dr. Maisch, Ammerbuch-Entringen, Germany) and after flushing from the trapping column the peptides were separated on a 50 cm µPAC™ column with C18-endcapped functionality (Thermo Fisher Scientific) kept at a constant temperature of 50°C. Peptides were eluted by a stepped gradient from 98% solvent A’ (0.1% formic acid in water) to 30% solvent B′ [0.1% formic acid in water/acetonitrile, 20/80 (v/v)] at 32 min up to 55% solvent B′at 43 min, followed by a 5 min wash reaching 70% solvent B’, all at a stepped flow rate starting from 750nl/min for 9 min to 300 nL/min, till the end of the run.

The mass spectrometer was operated in data-dependent, positive ionization mode, automatically switching between MS and MS/MS acquisition for the 5 most abundant peaks in a given MS spectrum.

The source voltage was 3kV, and the capillary temperature was 275°C. One MS1 scan (m/z 400−2,000, AGC target 3 ×E6 ions, maximum ion injection time 80 ms), acquired at a resolution of 70,000 (at 200 m/z), was followed by up to 5 tandem MS scans (resolution 17,500 at 200 m/z) of the most intense ions fulfilling predefined selection criteria (AGC target 50.000 ions, maximum ion injection time 80 ms, isolation window 2 Da, fixed first mass 140 m/z, spectrum data type: centroid, intensity threshold 1.3xE4, exclusion of unassigned, 1, 5-8, >8 positively charged precursors, peptide match preferred, exclude isotopes on, dynamic exclusion time 12 s). The HCD collision energy was set to 25% Normalized Collision Energy and the polydimethylcyclosiloxane background ion at 445.120025 Da was used for internal calibration (lock mass). QCloud was used to control instrument longitudinal performance during the project (18).

#### Data analysis

The resulting MS/MS spectra were analyzed with the BioPharma Finder 5.2 software (Thermo Fisher Scientific) and mapped onto the appropriate protein sequence. For peptide identification, the following parameters were used: maximum peptide mass of 12,000 Da, mass accuracy of 10 ppm and a minimum confidence of 0.50. Cysteine carbamidomethylation was set as a variable modification. The maximum number of variable modifications per peptide was set at 1.

### Peptides for antibacterial activity assays

Mature peptides for parental defensins Defa4 (a positive control), Defa17, Defa24, Defa30, Defa31 and Defa35 and chimeric peptides Defa17-24, Defa30-31 and Defa35-30 were custom ordered for synthesis from Life Tein custom peptide synthesis service (www.Lifetein.com). Proteins were aliquoted in PBS to a stock solution of 1 mg/ml and thawn when necessary, just once.

### Anti-bacterial activity assays

Antimicrobial essays for the synthetic proteins were performed against *Escherichia coli*, *Staphylococcus nepalensis*, *Enterococcus fecalis* and *Citrobacter rodentium*. The first three species are common in small intestine in mice (where Paneth cells are relevant), while *C. rodentium* is a common pathogen in mice. To obtain cultures of the first three species, C57BL/6J mice were euthanized, ileum content harvested and diluted in sterile PBS and plated out on standard LB plates, overnight. Colonies were picked and species identified using Maldi-Toff [as previously described (19)]. The relevant pure colonies of these three species were then grown on plates, identified once again, and grown in liquid LB and frozen at -80°C. The *C. rodentium* culture was strain ICC 169 and was obtained from ATCC.

Bacteria were grown in LB broth at 37°C in an incubation shaker (100rpm) until and OD between 0.8-1. For each peptide, a dilution set (800 µl/peptide) derived from the stock solution was prepared in such manner to obtain a final volume of 1 ml with peptide concentrations of 20 µg/ml, 10 µg/ml, 4 µg/ml and 2 µg/ml. Bacteria grown in LB broth where diluted to OD 0.2. For each bacterium, 200 µl of the OD 0.2 solution was added to each of the peptide dilutions and incubated at 37°C for four hours at 55 rpm. After incubation 100 µl of the incubated mixture was plated out on LB agar plates at two concentrations: undiluted and 1:10 diluted. The plates were incubated at 37°C overnight after which colonies per plate were counted and tabulated. The highest plated out concentration that still had individually distinguishable colonies was used.

The R package “drda” (20) was used to perform a dose-response analysis using the logistic 4 parameter model. The concentration that inhibits growth by 50%, IC_50_, was obtained from the models for each combination of bacterium and peptide and converted into per peptide specific activity.

### Statistics

All statistical analyses were performed in graphpad prism 9 except for the or R. Large scale genome wide analyses expression analyses and the anti-bacterial activity assays preformed using R and the DESeq2 and drda libraries. All other analyses were performed with graphpad prism.

### Study approval

Animal work performed directly for this manuscript did not involve procedures requiring ethical approval. All data GEO sources included in the manuscript had ethical approval from the Ethics Committee of VIB/UGent-Faculty of Science (Ghent University).

### Study graphical overview

In order to provide an overview of the workflow performed in this work we have created an overview flowchart figure as **Supplementary Figure 3**.

## Results

### The α-defensin locus

**Figure 1:**
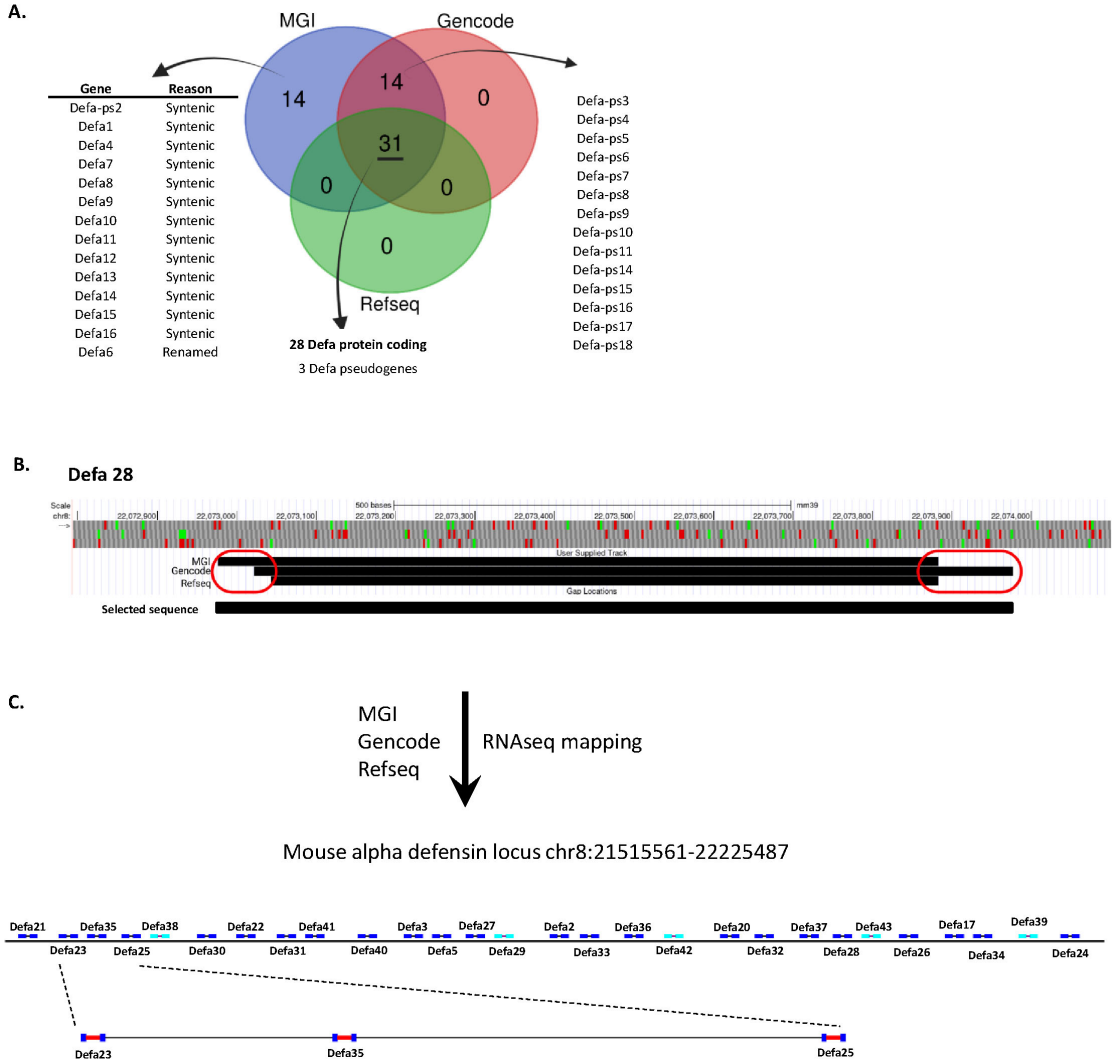
Update of the annotation of the *Defa* locus. **(A)** Number of annotated genes per Defa category and annotation source. **(B)** Visualization of an example (*Defa28*) of the variations in start and stop sites of *Defa* genes across the different annotation sources. **(C)** Construction and structure of the final *Defa* locus, based on current data, including RNA-seq, and further used in the analyses of this manuscript.

The α-defensin locus contains numerous genes and pseudogenes, the amounts of which is depending on the genomic annotation database that is used. In this study, we used three primary sources: MGI (15), RefSeq (13) and GENCODE (14), to obtain an overview of the actual number of α-defensin genes. After comparing the 3 annotation databases, a core set of 31 genes was found to be shared: 28 protein-coding genes and 3 annotated pseudogenes. Both MGI and GENCODE, which are less strictly curated than RefSeq, contain an additional 14 pseudogenes. Furthermore, the MGI set also includes an extra 14 genes that are annotated as “syntenic”: these genes lack a genomic location in the current genome annotation and should be considered historical records. Finally, MGI uniquely annotates one gene, *Defa6*, which is absent in the other datasets (**Figure 1A**).

The core set of shared genes rarely show perfect alignment across the different annotations. In most cases, there are minor differences of a few base pairs between the start and/or stop locations from each source (see for example *Defa28* in **Figure 1B**). However, more substantial differences are occasionally observed, with the largest discrepancy being a change of 112 base pairs. Given that the total length of an α-defensin gene is around 900–1000 base pairs, this represents a significant difference in both location and gene size. It is important to keep in mind that all annotations contain full length coding sequences of the genes, and that the observed differences are limited to the untranslated up- and downstream sequences, except in case of *Defa6*/*Defa24*, explained below. However, taking the maximal possible size for genomic location will allow more reads to map to the genes, especially in cases where the size increase is substantial.

The largest discrepancy occurs with *Defa6* and *Defa24.* In both RefSeq and GENCODE, *Defa24* is considered as the current name for the gene that was previously annotated as *Defa6*. However, this is not fully clear: *Defa6* does have a RefSeq record, but the sequence associated with this record cannot be found in the genome. The closest hit, with two mismatches, is *Defa24*. In addition, the *Defa6* record in the RefSeq annotation accessible on the UCSC genome browser (and others) redirects *Defa6* to *Defa24*. In MGI however, both *Defa6* and *Defa24* exist, with *Defa24* matching the annotation in RefSeq and GENCODE and with *Defa6* being located immediately in front of *Defa24* (MGI *Defa6*: Chr8:22,223,899-22,224,507; MGI/refseq/GENCODE *Defa24*: Chr8:22,224,510-22,225,487) at the time of writing. Since *Defa6* is only listed in MGI, it is not included in the core set of 28 protein-coding genes, whereas *Defa24* is. Based on our RNA-seq data, *Defa6* is non-existing as a separate gene, and is indeed the old name of *Defa24*.

For subsequent analyses on the core set of the 28 *Defa*-coding genes, by default, we considered the earliest possible start and the latest possible end positions of the transcripts, even if these were derived from the different annotation sources. We used the GENCODE GTF as a basis and updated all records for these 28 genes. This was done to maximize the sequence that could be used to quantify the transcript in later steps. Where possible and applicable, we utilized actual mapping data from our own RNA-seq datasets, generated through deep, bulk RNA-seq of purified, sorted Paneth cells, as described later. As an example, we illustrate the transcript sizes and positions of the *Defa28*-coding gene across the three databases: in this case, we observe three different start sites and two distinct end sites. For all analyses in this work, we use the start site from MGI and the end site from GENCODE as this yields the longest gene (**Figure 1B**).

Over the previous years, we have sorted and purified Paneth cells from mouse small intestines via FACS and performed RNA sequencing [see **Supplementary Figure 1** for the sorting strategy, as described in Methods and in (12)]. As recently described by us (12), the sorted PCs are 99.9% pure, and a single C57BL/6J mouse on average yields some 25,000 PCs. We thus obtained RNA-seq data comprising over 30,000 different transcripts per sample and millions of reads. In total, we sequenced 148 samples of ± 25,000 cells from individual mice, using various treatments and technologies (including single- and paired-end sequencing, Illumina NovaSeq, NextSeq, and Element AVITI). For this study, we mapped the reads to the mm39 mouse genome with the updated GENCODE reference and used our mapped data to further refine the updated annotation.

Based on our analysis, we made a graphical representation of the α-defensin locus, which will be applied throughout this study. This structure excludes pseudogenes (**Figure 1C**). The intergenic distances are drawn to scale, while the gene representations are inflated for clarity. 23 genes are located on one strand while 5 genes are on the opposite strand. To have an estimation of sizes of the genes and the intergenic distances, we zoomed in on 3 genes which are drawn to scale. Also obvious is the typical 2-exon structure of all *Defa* protein-coding genes.

### The α-defensins in mice and other species

The mouse genome has 28 protein-coding α-defensin family genes. This extensive gene count in the locus is mainly the result of tandem duplication events and is much greater than found in several other well-known species, including humans. To determine whether the large number of α-defensin genes is unique to mice (*Mus musculus*) or shared across multiple species, we conducted a comparative analysis with several other species. The species were selected based on three criteria: (i) evolutionary distance, including both closely and more distantly related species, (ii) the cellular location of α-defensin expression: some species express these genes exclusively in intestinal Paneth cells [e.g. mouse (21)], while others express them in both Paneth cells and neutrophils (e.g. *Homo sapiens*), and (iii) the availability of data.

For the comparison with the mouse, we included the following species: Algerian mouse (*Mus* sp*retus*), brown rat (*Rattus norvegicus*), Chinese hamster (*Cricetulus griseus*), Guinea pig (*Cavia porcellus*), naked mole-rat (*Heterocephalus glaber*), rabbit (*Oryctolagus cuniculus*), horse (*Equus ferus caballus*) and human (*Homo sapiens*). Of these, only the horse expresses α-defensins exclusively in PCs, like mice (22). Although pigs (*Sus scrofa*) and sheep (*Ovis aries*) are also reported to have Paneth cell-specific α-defensin expression (22), no genes of this type were found annotated or predicted in the current genome versions, so they were excluded from the analysis.

**Figure 2:**
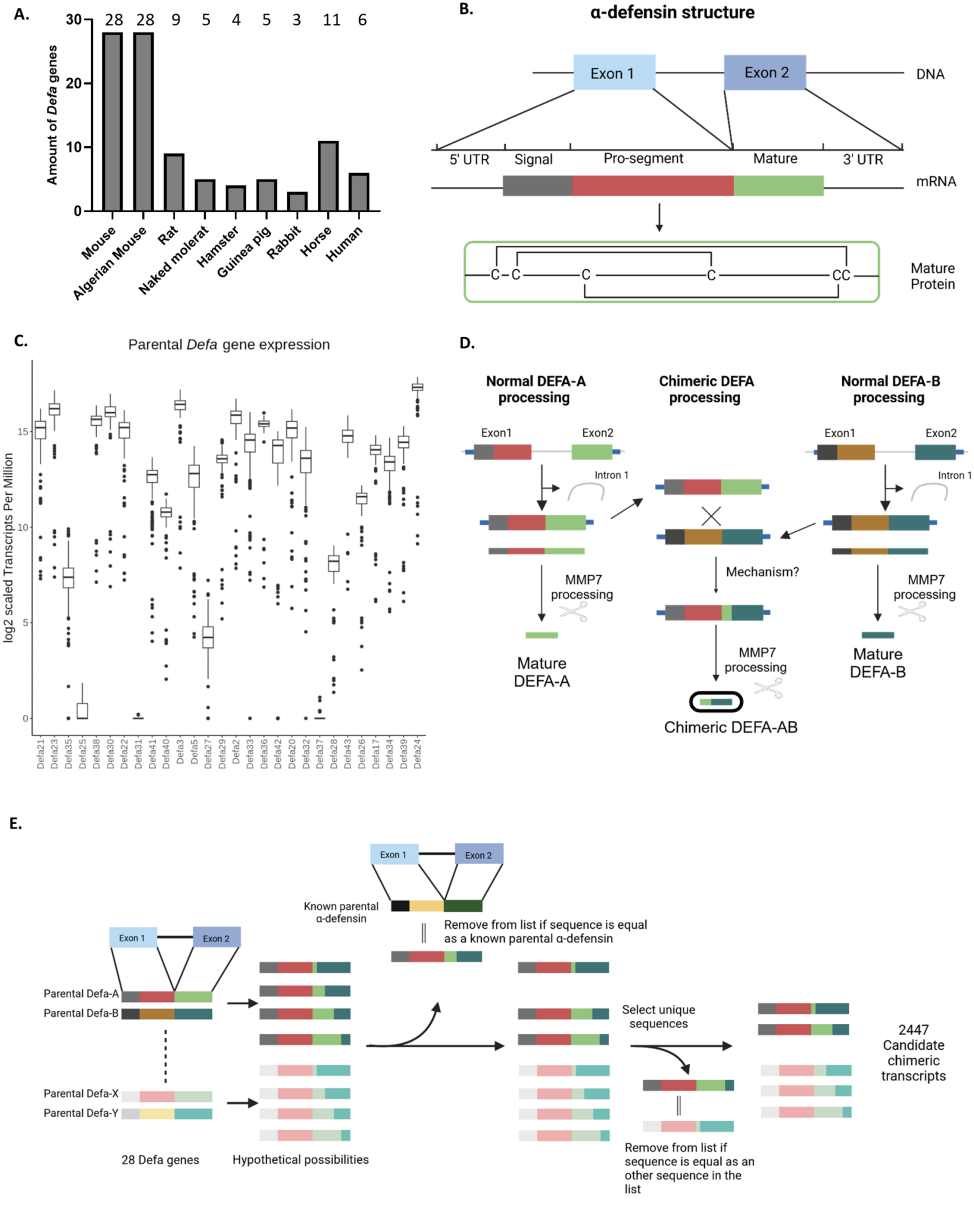
α-defensins in mice and other species and chimeric mRNAs. **(A)** Amounts of α-defensin genes found per species in this study. **(B)** General structure of an α-defensin gene, mRNA and final biologically active peptide. **(C)** Expression levels of all 28 *Defa* genes in mouse Paneth cells, based on RNA-seq data in a box plot. Values are expressed in tags per million (TPM), as explained in Results. **(D)** Cartoon displaying how canonical *Defa* gene mRNAs and proteins are formed, and how chimeric Defa mRNAs and proteins look like. **(E)** Schematic workflow of the construction of the theoretical chimeric defensin sequences.

We evaluated species-specific annotated α-defensin genes where available (*e.g.* mouse, rat and human). In species in which α-defensins were not directly annotated, we relied on *in silico* predictions, available in genome browsers, such as the UCSC genome browser (23). These predictions were extensively filtered, as described in the Methods section. Briefly, predictions were filtered on gene size, gene structure and overlapping regions were collapsed into one. This process resulted in a final set of a handful of genes per species, compared to the often many dozens of predictions. All species compared to the mouse, except the Algerian mouse (estimated to have separated from *Mus musculus* 1 million years ago (24, 25), have much less α-defensins. Only the rat, which is evolutionary the closest related to mouse, and the horse, which share the same cell type expression pattern as mouse, have slightly more α-defensin genes, with 9 and 11 genes respectively. All other species have between 3 and 5 α-defensins, indicating that *Mus musculus* and *Mus* sp*retus* are outliers in this regard (**Figure 2A**). We cannot exclude that the low amount of defensins found in other species is a detection/annotation or sequence assembly problem or actually cause by biological process. For humans, which also have a well-studied genome, we are confident that the genes detected and reported are indeed accurate. He rat genome is less studied than the mouse one, and there has been no extensive study and consolidation of the locus as was performed in mice by, among others, Amid et al., so it is possible that the number of rat α-defensins is underestimated. The other species are primarily/exclusively *in silico* predictions and remain a best guess and an exhaustive study and annotation is beyond the scope of this work.

### Expression of α-defensins

Transcript quantification was performed using Salmon, as described in the Methods section, and was based on transcripts from the updated α-defensin locus (**Figure 1C**) as well as GENCODE transcripts for all other transcripts across the 148 samples. The typical α-defensin gene structure (**Figure 2B**) consists of a small gene with 2 exons. The high sequence similarity between these genes makes read-assignments challenging. In both the previous STAR mapping and Salmon quantifications, we observed the following: (i) a lower mapping rate than expected, accompanied by a high number of multi-mapped reads, (ii) a higher base mismatch rate than expected considering the sequencing quality and from the STAR mapping data, (iii) the occurrence of what appeared to be “fusion or chimeric transcripts/genes”, supported by reads mapped to two different genes as junction spanning.

The gene abundance results that were obtained by the best-possible mapping, without taking the fusion transcripts into account, revealed uneven expression levels across the α-defensin locus. Since each of the 148 bulk RNA-seq experiments yielded different amounts of cells and counts, in each of these experiments, the total amounts of mRNA counts was normalized using the tags per million (TPM) method.

In **Supplementary Table 1**, we display the RNA-seq results of a typical experiment, in which three C57BL/6J mouse Paneth cell samples were sequenced and the data pooled and normalized to a total of 1 million reads (the actual amounts of reads was around 25 million). In this experiment, we detected 69.737 unique transcripts. In the 20 most abundant transcripts, there are 18 *Defa* genes, one is *Lyz1* and one is *Intel1*. In the second tab of the list, we display the Tags Per Million of the *Defa* genes. As usual, we were unable to find reads for *Defa31* and *Defa37*. The total amount of *Defa* TPM in this experiment was 851.910, thus 85.19% of all mRNAs in Paneth cells (in this experiment) were from *Defa* genes.

**Figure 2C** represents the amounts of counts of each *Defa* transcript in a box plot, expressed in TPM. With 2^17.327^ (=164,417) counts/million*, Defa24* is the most highly expressed gene. Others, such as *Defa25*, showed minimal expression across the samples. Interestingly, these gene expression patterns did not correlate with genomic location or orientation, as illustrated in **Figure 2C**, where the genes are displayed in genomic order in the box plot.

Many different chimeric transcripts, i.e. mRNAs containing sequences of two (rarely more) different *Defa* genes were observed. Further investigation into these “fusion transcripts” detected during mapping with STAR and confirmed to be also found with another mapping tool (HISAT2), revealed several cases in which the biologically active part of the mature peptide (coded by the second exon) (**Figure 2B**) would be affected. A cartoon, summarizing the types of detected events, is shown in **Figure 2D**. Overall, we observe that (any) two α-defensins are transcribed into their respective mRNA molecules, which are detected by RNA-seq. It can be safely assumed that these transcripts will eventually be translated and processed into their final active peptide form. In addition, we also identified chimeric mRNAs that contain sequences from both parental genes within their second exon. This fusion creates a unique sequence, leading to the production of a novel (active) peptide, distinct from the peptides encoded by both the parental genes.

The high sequence similarity between the α-defensin genes makes the pinpointing of the exact location of chimerization events challenging. Furthermore, there are indications that multiple chimeras may be formed from the same parental gene pair through different chimerization sites. To investigate the potential chimeras, we generated a set of theoretical chimeras whose existence could then be confirmed or disproved by the available *in vivo* RNA-seq data as described in methods. Briefly, for each pair of parental combinations, all possible theoretical chimer sequences were created as cDNA sequences per donor-acceptor pair. Restrictions on frameshifts, early stops, and sequence similarity to parentals and other potential chimeric sequences derived from the same pair were immediately taken into account during the generation. Afterwards the sequences from all pairs were pooled and further filtered by comparing them to all known α-defensin cDNAs, and all sequences identical to known α-defensins were removed from the dataset. Next, we compared the remaining generated sequences to one another and collapsed duplicates, retaining only one occurrence per sequence. This resulted in a final set of 2447 candidate chimeric transcripts (**Figure 2E**).

### Detection and abundance of chimeric transcripts

**Figure 3:**
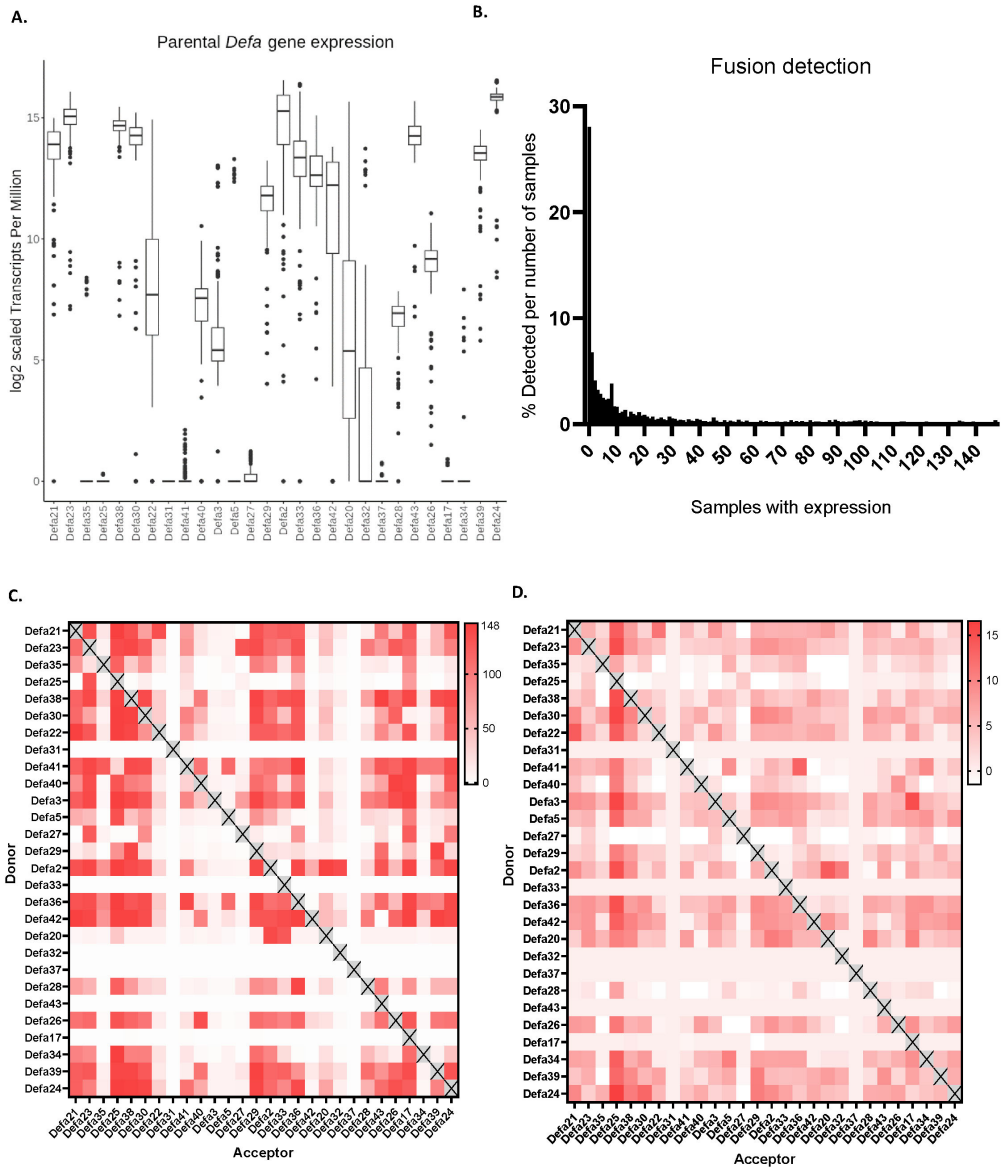
Aspects of theoretical and observed chimeric mRNAs. **(A)** Effect of the inclusion of theoretical chimeric mRNAs on the detection of parental mRNAs. **(B)** Histogram of how many (% of 2447) theoretical chimeras are detected in function of the number of sequenced RNA-seq samples. **(C)** Occurrence heat map of the observed chimeric mRNAs. In the plot, the color represents the number of RNA-seq samples in which chimeric transcripts are found, the maximal amount being 148. **(D)** Abundance heat map. Over 148 bulk RNA-seq experiments, in which all reads are normalized to 1 million in each experiment, chimeric mRNAs usually show between 5 and 10 TPM reads.

After incorporating the theoretical chimeric transcripts to the observed set of transcripts from RNA-seq, we generated three additional Salmon indices: (i) one that included both parental and chimeric sequences, (ii) one with parental sequences (the same as the standard transcript set) and chimeric transcripts as decoy sequences and (iii) one with chimeric sequences and parental sequences as decoys. The inclusion of the fusion transcripts has a large effect on the number of reads that could theoretically be quantified as well as the actual abundance detection of parental α-defensins (**Figure 3A**). The overall abundance of parental transcripts decreases, as expected, by involving the theoretical chimeric transcripts. In the case of *Defa24*, for example, the TPM decreases from 2^17.327^ to 2^15.863^ (i.e. from 164,417 to 59,599) and some show a much greater reduction than others. For instance, *Defa35* dropped from a median expression of 173.6 TPM to 0 TPM, with expression being detected in far fewer samples. An overview of all median TPM values of **Figures 2C**, **3A**, as log_2_, is shown in **Supplementary Table 2**.

We then studied how many of the 2447 theoretical chimeric sequences we actually found across our 148 RNA-seq samples. We set these theoretical cases that we found as 100% and plotted the distribution of these as function of the amounts of RNA-seq samples. The largest group is made of sequences that were never found in any of our samples, a total of 687 (28%) of chimers falls in this category. Overall, the distribution is heavily shifted to the left with a long right tail, similar to a power law distribution. A total of 1987 (81.2%) of the sequences can be found in one third of the samples or less. If the never found sequences are excluded, we find 1300 sequences that are found in at least one and at most 50 samples, which make up 53.13% of the total number of sequences (or 73.86% of the detected sequences). In contrast only 7.8% of the sequences (191) are found in at least two thirds of the samples (>100) (**Figure 3B**).

Not all *Defa* parental genes participate equally in forming chimeras, as found in our 148 RNA-seq samples. When all donor and acceptor combinations were studied *per* parental pair, we found several that form at least one chimera in all samples. In **Figure 3C**, we plot the occurrence of all observed chimeric mRNAs in a heat map. The color represents the number of RNA-seq samples in which chimeric transcripts are found, the maximal amount being 148. There is a clear bias for several defensins to act more as donors or as acceptors. For example, *Defa25* and *Defa32* predominantly function as acceptor parents, forming more chimeras in this role than as donors, while *Defa41* exhibits the opposite behavior. Interestingly, some α-defensins, such as *Defa31 or Defa37*, are almost never found to be part of any chimeras. However, it is challenging to determine whether this is due to biological reasons, and that they do not form chimeras, or technical limitations, such as reads that cannot be mapped to these sequences preventing their detection.

Finally, the abundance of the chimeric mRNAs is plotted in **Figure 3D**. As in **Figures 2C**, **3A**, the TPM is plotted, this time in a heat map. Over 148 bulk RNA-seq experiments, in which all reads in each experiment are normalized to 1 million, the observed chimeric mRNAs remain quite low in expression, usually between 5 and 10 TPM, with a maximum of 16.6 TPM. The actual TPMs are found in **Supplementary Table 3**. As an example, *Defa24* sequences are found in chimeric mRNAs as donor in 160.5 copies per million transcripts, while as acceptor in 86.0 copies per million, thus in total in 246.5 copies per million. Compared with the maternal *Defa24* copies, this is low indeed. Such median numbers apply only for the number of samples in which the chimeric mRNA have actually been detected (in the 148 datasets). Across all the *Defa* genes, the total amount of mRNAs per million involved as donors (as well as acceptors) in chimeric sequences is 2413 per million. This is 0.24%, and thus quite limited. On the other hand, if we would combine all these chimeric Defa mRNAs and add them to the list of the RNA-seq example of **Supplementary Table 1**, they would occupy the 28^th^ place of most abundant mRNA in total.

### Detection of chimeric proteins

**Figure 4:**
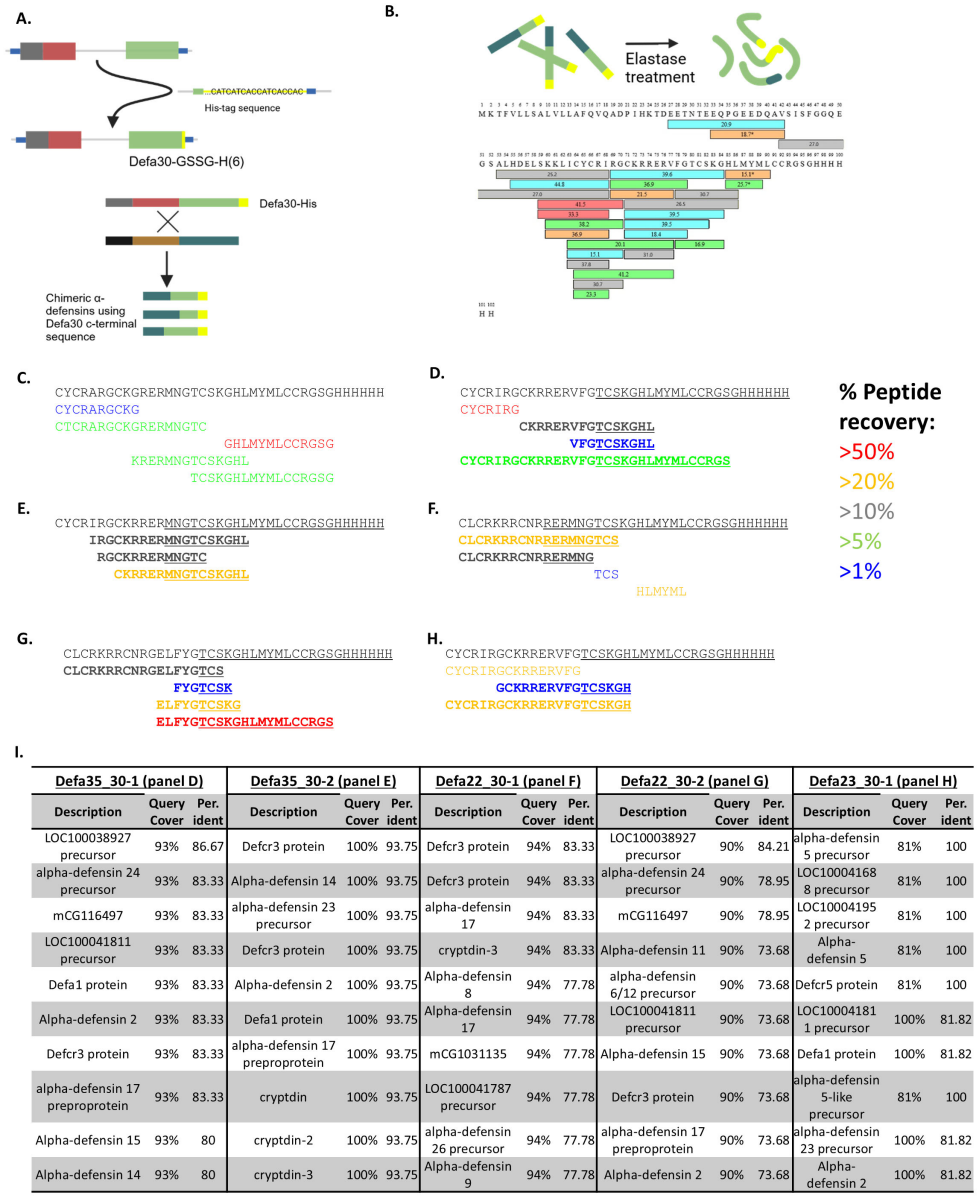
Detection of chimeric α-defensin peptides. **(A)** The principle of adding a His6 tag to the C-terminal part of Defa30 protein via Crispr/Cas9 mutagenesis in the mouse genome, the Defa30 His-tagged protein and schematic representation of potentially tagged chimeras. **(B)** Mixture of His-tag enriched proteins after purification and digestion with elastase. The sequence of Defa30His6 protein is shown, as well as the peptides that were found by the MS analysis. The different colors of the peptides reflect their reliability (red>orange>gray>green>blue). **(C)** parental α-defensin peptides recovered, completely matching Defa30 protein and gene. **(D, E)** Peptides recovered for 2 theoretical chimeras of Defa35 and Defa30. Peptides in bold are those that span the chimeric region and support the chimerism. **(F, G)** Peptides recovered for 2 theoretical chimeras of Defa22 and Defa30. Peptides in bold are those that span the chimeric region and support the chimerism. **(H)** Peptides recovered for a theoretical chimera of Defa23 and Defa30. Peptides in bold are those that span the chimeric region and support the chimerism. **(I)** Blastp results of chimerism region peptides from **(D–H)**. There is no match with any genomic encoded protein in the mouse non redundant protein database.

After identifying chimeric α-defensin mRNA in the RNA-seq data, we aimed to confirm the presence of actual chimeric proteins *in vivo*. Due to the very small size of α-defensins they are challenging to purify, and the expected low abundance of chimeras further increases the difficulty of detecting them. For this reason, we generated transgenic mice in which the *Defa30* gene was replaced with a His-tagged version (His6 tag, see Methods). The reason of selecting Defa30 for that matter was based on the facts that (i) the *Defa30* mRNA appeared to be selected as an acceptor in the formation of chimeric mRNAs and (ii) the genomic sequences surrounding the *Defa30* gene were compatible with specific CRISPR/Cas mutagenesis, given the very high sequence similarities found in the *Defa* locus. The tagged *Defa30* gene should result in the production of C-terminal His-tagged Defa30 protein as well as tagged chimeric proteins that have Defa30 as the acceptor parent (**Supplementary Figure 2**, **Figure 4A**). To detect these proteins, Paneth cells were sorted, lysed and the His-tagged proteins purified by standard techniques. These were fragmented using elastase, followed by separation and detection with mass spectrometry, and the full-length, canonical Defa30 peptide was recovered, as shown in **Figures 4B, C**.

Several translated peptide sequences of the predicted chimeric protein, with Defa30 as the acceptor parent, along with the entire mouse UniProt proteome were used as reference for sequence identification. **Figure 4C** shows the active part of the Defa30 protein sequence and several peptides that were matched to it. We went into the details of the proteomics results of two chimeric peptides from the Defa35 and Defa30 parents (**Figures 4D, E**), two chimeras from the Defa22 and Defa30 parents (**Figures 4F, G**) and one chimeric peptide from the Defa23 and Defa30 parents (**Figure 4H**). See **Figure 4I**.

For each of these chimeric sequences, we recovered peptides that not only matched them, but also spanned over the transition region of the two parents. To confirm that these transition-region-spanning peptides were indeed unique to chimeric α-defensins, a protein blast (blastp) against all mouse-specific protein sequences in the non-redundant protein database was performed. For each chimera, we selected the longest transition-region-spanning peptide. The blast results showed that none of the selected peptides completely matched any (mouse) protein present in the non-redundant protein database (**Figure 4I**). This indicates that the sequences detected in the proteome analysis can only be derived from chimeric sequences and are not encoded by any known mouse gene in the genome. These findings prove that chimeric α-defensins are not only transcribed but also translated into proteins within Paneth cells of mice, proving that the chimerization events do occur *in vivo*.

These chimeric Defa30 acceptor peptides were also supported by the RNA-seq read data in most cases. The high sequence similarity makes read mapping challenging, which is why we opted for a proteomics approach for additional confirmation, but several reads do support the chimeric region of the sequenced peptides. As a specific example, the Defa23-Defa30 chimer will be used (**Figure 4H**). On the peptide level, this chimer has 3 recovered peptides that span the chimeric region. On the RNA level the situation is more complex, as the reads are on average 60-80 bp long, and the difference in mapping fully to Defa23 or Defa30 is often 1 to 2 mismatches. We do find several reads that may be assigned to Defa23 and Defa30 in a manner that is consistent with the chimer. Specifically, we recover several reads with sequence (and different per base qualities) over the different experiments: “ATTAAATGACCCTTACTGCAGGTCCCATTCATGCGTTCTCTTCTTTTGCAGCCTCTTGTTCTACAATAGCA”, which cannot be mapped with 100% identity anywhere in the genome or transcription. However, the sequences does match with one mismatch in the second half to Defa23 and with 2 mismatches in the first half to Defa30 (**Supplementary Figures 4**, **5**, respectively). In conclusion the Defa23-Defa30 chimer that was detected in the proteomics is directly supported by the RNA-seq read data.

### Biological activity of chimeric α-defensins

Given the high sequence similarity between the classical α-defensins and chimeric α-defensins, we considered that they would show similar degrees of biological activities. To test this, we had maternal and chimeric peptides synthetized and tested their biological activity. We used classical α-defensins (Defa4 as a positive control, Defa17, Defa24, Defa30, Defa31, Defa35) and some of their corresponding chimeric versions (Defa17-24, Defa30-31, Defa35-30). There was no specific reason to select these defensins and chimeras, besides that they appeared possible to synthetize from a technical point of view. Dose-response analyses (see Methods) were performed to evaluate the inhibitory potential of the parental and chimeric α-defensins against growth of four bacterial species (*E. coli, C. rodentium, S. nepalenis and E. fecalis*). The specific activities (U/mg peptide) were determined by calculating the IC_50_ values for each peptide. We found that the activities of the peptides vary quite widely depending on both the bacterial species and the specific peptide tested. Both classical α-defensins and the chimeric peptides showed similar effects on bacterial growth. In two cases, the chimeric peptide exhibited intermediate biological activity compared to both parents, while in one case (Defa35-30), the antibacterial activity had stronger activity than both parents (**Table 2**). In conclusion, we were able to confirm that the chimeric peptides indeed contain biological activity. In one of the cases, the chimeric peptide shows superior activity.

**Table 2:** Specific activities of the cell killing activities of alpha defensin peptides.

Peptide	*E. coli*	*C. rodentium*	*S. nepalensis*	*E. fecalis*	Mean
Defa4	781	314	352	135	396
Defa17	438	402	332	395	392
Defa24	122	77	8	23	58
Defa17-24	312	361	359	193	306
Defa30	186	107	6	52	88
Defa31	397	314	437	189	334
Defa30-31	140	132	125	49	112
Defa35	262	260	77	127	182
Defa30	186	107	6	52	88
Defa35-30	626	774	372	269	510
Mean	348	289	245	148	258

As described, the peptides were titrated on bacterial cultures, and the antibacterial activity expressed as U/mg, whereby 1U is the dose needed to kill 50% of the bacteria (IC50). The data are means of two independent experiments.

## Discussion

In our database of unrelated bulk RNA-seq experiments on purified Paneth cells, some previously published by us, we observed peculiar events at the mouse α-defensin locus. The findings pointed to unexpected transcriptional events occurring at this gene region. Upon closer examination, it appeared that non-canonical mRNA transcripts were being produced, consisting of sequences from multiple genes.

We further explored these findings by re-aligning our RNA-seq data using STAR with identical settings across all samples, to closely examine the mapping results. To obtain maximal information, we combined annotation data from multiple sources (GENCODE, RefSeq, MGI) and investigated possible discrepancies between them. Despite previous efforts in curating the locus (26), we did not find perfect agreement between the sources. While overall agreement in coding gene annotations is string, there are notable differences in the quality of UTR region annotations. The *Defa6* gene annotation in MGI has also been investigated for its relevance as the corresponding sequence could not be found back in the genome and is likely an error in this database.

Based on current annotations, mice appear to be an outlier species, with an unusually large number of α-defensin genes that are not present in any other species, including closely related rats (26). In fact, both the house mouse (*Mus musculus*) as well as the Algerian mouse (*Mus* sp*retus*), two distinct species that separated an estimated 1 million years ago (24, 25, 27, 28), have 28 protein coding *Defa* genes in their genome. But the data may indicate that mice are less suited as a model for α-defensin related research, or that mice have undergone gene duplications under environmental pressure, and well after the evolutionary split from rats, or that additional α-defensin genes remain to be discovered in other species. This latter possibility cannot be ruled out, as we only detected chimeric reads and high α-defensin transcript counts when analyzing purified Paneth cells and it has been shown that α-defensins in vertebrates evolve very rapidly with rapid increase in gene number and strong positive diversifying selection (29). In other species, Paneth cell purification followed by bulk RNA-seq has not been previously performed, and research typically relies on organoids or small intestinal biopsies for analysis (30). Additionally, human and pig biopsies are mostly analyzed at the single-cell level, which provides less depth than bulk RNA sequencing. Even if not, the very low amounts of material from such a biopsy prevent Paneth cell enrichment. Therefore, it is important to note that the extended number of genes and especially the chimeric events in RNA-seq studies of ileal biopsies or single-cell RNA-seq might completely escape detection.

In mice, by bulk RNA-seq, a wide variety of α-defensin related mapped reads were recovered, including those indicating the occurrence of fusion transcripts. Since only events involving a change in the second exon of these genes would affect the final active peptide produced (5, 8), we focused solely on these events and excluded exon 1 events and trans-splicing. In addition, we did not include any events occurring in α-defensin pseudogenes. While this approach may cause us to overlook some pseudogene-related events, it was done to increase the biological relevance of the obtained results as well as to speed up the computational analyses.

The events provide a novel form of fusion gene transcript expression, next to what was previously described in literature. It has been known for many years that chimeric/fusion genes are often expressed in context of cancer and may indeed be drivers of carcinogenesis. However, in these cases it usually concerns genomic abnormalities, such as chromosomal translocations inversions that create the fusion gene in the genome itself (31–33). The non-genomic sources of chimeric transcripts in eukaryotes, both in and outside of cancer context, are trans-splicing and gene read-trough events. Trans-splicing is a process where chimer mRNAs are created in the spliceosome by joining exons of and is a well-documented process in vertebrates (34). Transcriptional read-through is a second described pathway that can give rise to chimeric transcripts. This mechanism is limited by gene proximity and restricted neighboring genes (35, 36). Over time this may give rise to novel genes if they provide an evolutionary advantage (35).

Despite the high sequence similarity, we successfully detected and quantified hundreds of chimeric transcripts from the α-defensin locus. However, since differences in read mapping can sometimes be as small as a single nucleotide, and given that sequencing is not error-free, we cannot rule out that our observation may be a sequencing and/or analysis artifact purely on mRNA level data (37). Since one of the datasets was the result of paired-end high fidelity RNA-seq (which also confirmed the reported events), we decided to use proteomic data to confirm the *in vivo* appearance of these chimeric peptides. The enrichment approach using a His6-tagged Defa30 transgenic mouse (Defa30His6^Tg/+^), along with elastase proteolytic processing, confirmed the existence of chimeric α-defensins at the protein level, at least those involving Defa30. Notably, several junction-spanning peptides were identified that could not have originated from any gene directly encoded in the mouse genome. Furthermore, we also proved that these chimeric α-defensins were indeed biologically active and exhibit (or maybe even surpass) effectiveness comparable to canonical α-defensins. However, there is significant variation in their efficacy depending on the sequence and bacterial strain.

The total amount of chimeric transcripts found in the RNA-seq experiments (in which the total amount of all transcripts is normalized to 1 million) is about 2.413 PTM. Since the total amount of all Defa genes taken together is in the order of 850.000 TPM, measured over all 148 samples, it follows that about 0.3% of the Defa mRNAs consist of chimeras. This is limited, but on the other hand, if we would combine all the chimeric Defa mRNAs and add them to the list of the RNA-seq example of **Supplementary Table 1**, they would occupy the 28^th^ place of most abundant mRNA in total. The total amount of Defa TPM in this experiment was 851.910, thus 85.19% of all mRNAs in Paneth cells (in this experiment) were from *Defa* genes and 0.3% from chimeric Defa mRNAs.

As the formation of a chimeric mRNA consumes a donor and an acceptor mRNA copy, the added value of producing some chimeric mRNA copies does not reside in quantitative aspects. One added value might be that chimeric defensins might be able to deal with more different bacteria than the mammalian genome is actually coding for, or that chimeric defensins are more powerful than the parentals, toward certain bacteria. Given the very high sequence similarities between defensin peptides, this increase in antimicrobial power appears hard to believe. Nevertheless, it did catch our attention, in the antimicrobial tests, that the chimeric Defa35-30 had higher specific activities in killing the four bacterial species that we tested, as compared to Defa35 or Defa30.

The mechanism by which the chimeric transcripts are created remains elusive. There is little evidence to match currently known mechanisms of action. There is no evidence for any genomic re-arrangements nor is there support for trans-splicing as described previously as the locations where the chimerism occurs vary and is not at all related to the splice sites. In addition, as previously mentioned, there is evidence that actual trans-splicing may occur, but without effect on the final peptide. Finally, since we observe many events from genomically distant genes, even if on the same locus and between genes on opposite strands, we also exclude transcriptional read-through as a potential mechanism.

Since we never observed chimeric mRNAs between any of the *Defa* mRNAs and other very abundant mRNAs in Paneth cells (*Lyz1* or *Itln1* for example), we believe that the mechanism is not simply due to abundances of mRNAs, but that mRNA sequences *senso stricto* must play some role. We investigated several possibilities without reaching conclusive results. The fact that multiple chimeras may be produced from any one parental pair does not support a sequence motif-based mechanism. Additionally, the high variability in the detection of chimeras and canonical α-defensins, along with technical noise and the lack of correlation between their abundances, further argues against a sequence-based or -driven mechanism. We also investigated the secondary mRNA structure trough predicted folding [ViennaRNA (38)], but again found no correlation between secondary structure formation and the frequency or location of chimer formation.

Based on all this information, we propose two hypotheses. The first possibility is a semi-active stochastic mechanism, where mRNA molecules from α-defensins, which are produced in immense quantities in Paneth cells, occasionally undergo random breakage and repair, thereby forming erroneous chimeras. Secondly, the massive production of (α-defensin) proteins in Paneth cells induces stress and activates the unfolded protein response (UPR). This is a process that is well-known and described in various secretory cells (11). The goals of the UPR in to help deal with misfolded proteins by up-regulating various folding proteins (e.g. heat shock proteins) to help with folding, moderate translation and transcription processes or promote degradation of proteins that cannot be rescued. The UPR may be activated by three main sensors: PERK, IRE1 and ATF6. In context of our hypothesis, IRE1 activation and activity is the most interesting. The IRE1 protein (coded by *Ern1 and Ern2* genes) is an endonuclease responsible for processing the *Xbp1* mRNA into its “spliced” form, Xbp1-s, by excising a cryptic intron, which in fact has no resemblance to classical splicing (splice acceptor, splice donor) at all (39). We propose that IRE1 may show a low amount of off-target activity in Paneth cells, acting at the level of α-defensin mRNA molecules, which may be cut and pasted. Regardless of the cause of breaks, erroneous repair ligation might occur by an ER ligase such as RtcB (40).

The added value of the existence of the chimeric defensin peptides is not clear, and as long as we found no way to generate mice which can no longer form the chimeras, will remain pure speculation. One of the chimeric peptides that we synthetized appeared to have better antimicrobial activities than the parentals on all four bacterial species, but a future study involving all possible hundreds of different chimeric peptides that we detected should be performed to draw general conclusions on this aspect.

In conclusion, we have proven the production of non-canonical α-defensin mRNA molecules at both the RNA and the protein level. The RNA evidence shows that chimeric α-defensin is a wide-spread, but not universal, phenomenon among all α-defensin genes at low amounts. The strong protein level evidence, with several chimeric region-spanning-peptides, proves that the mRNA observations are not artifacts from sequencing or data processing. Unfortunately, the proteomics data did not allow for quantification of the proteins, but only identification. We also propose potential mechanisms of how these chimeras might be produced, but this will require extensive follow-up work to elucidate.

## Data Availability

The datasets presented in this study can be found in online repositories. The names of the repository/repositories and accession number(s) can be found in the article/**Supplementary Material**.
