# Supplementary material for: Detection of chimeric alpha-defensin transcripts and peptides in mouse Paneth cells
Source: Front Immunol. 2025 Feb 6;16:1543059. doi: 10.3389/fimmu.2025.1543059 (PMC11840258; doi:10.3389/fimmu.2025.1543059)
Supplement: Supplementary file 1 [file DataSheet1.pdf]

Supplemental Figure 1

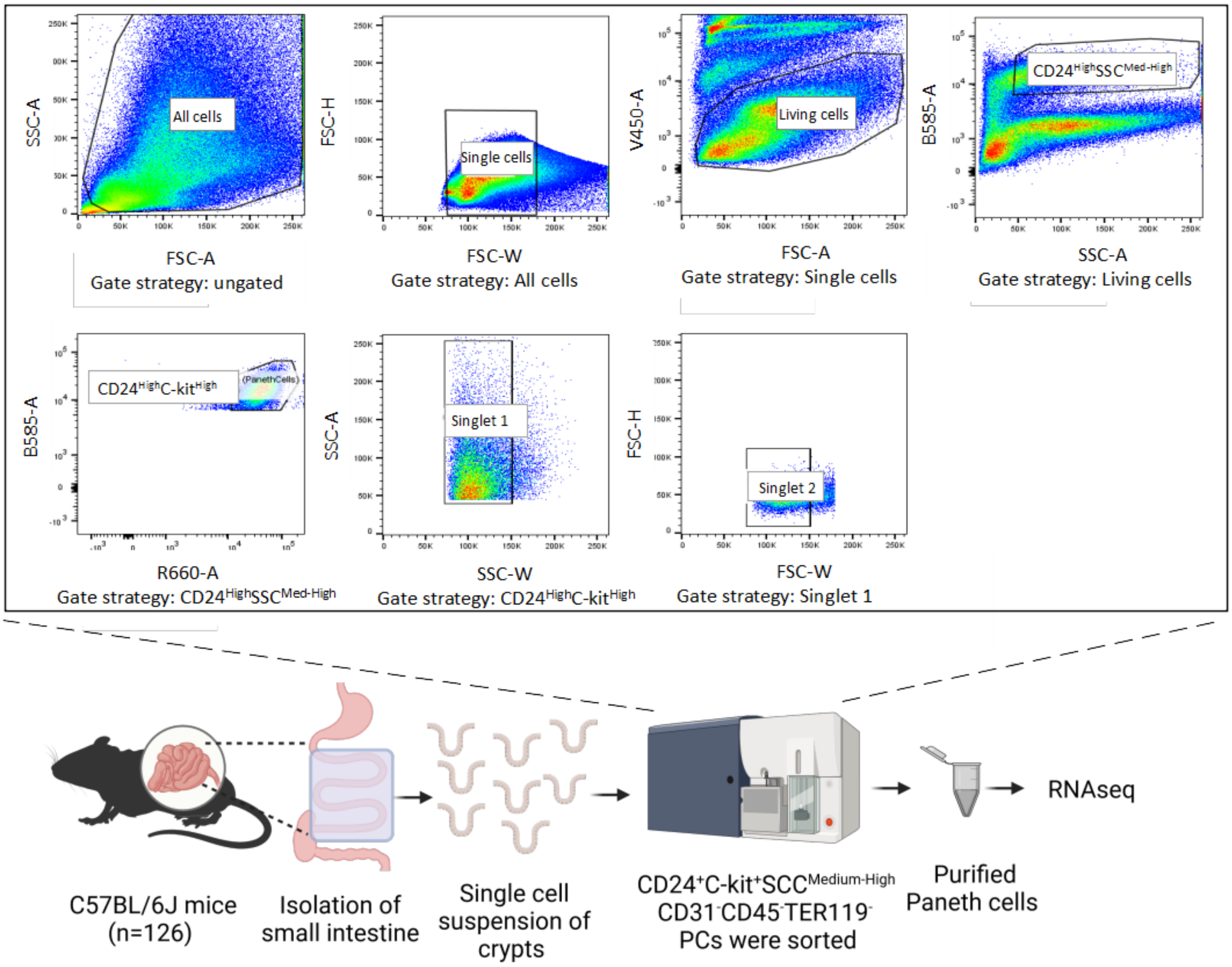

Supplemental Fig 1. Workflow of the RNA-seq experiments. The small intestine of C57BL/6J mice was isolated, crypts were purified and Paneth cells sorted via FACS using the markers mentioned and pooled to obtain about 25,000 PCs using the protocol published in (Timmermans *et al*, 2024). Cells were sequenced with illumina sequencing device or Element AVITI

Supplemental figure 2

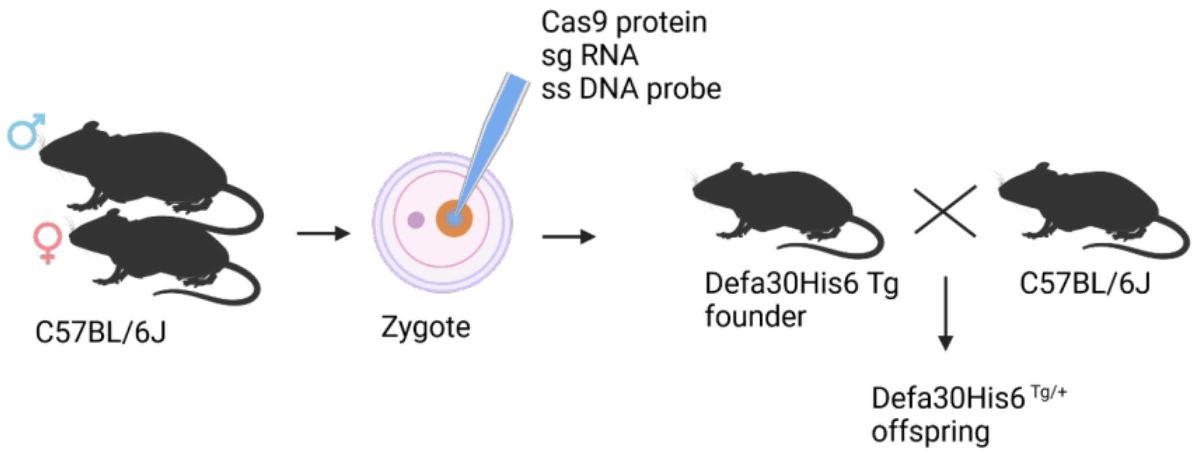

Supplemental Fig 2. Schematic representation of generation of Defa30 mutant mouse. Male and super-ovulated female C57BL/6J mice were crossed and zygotes isolated. These were electroporated with a mixture containing Cas9, a guide RNA and a ssDNA probe. Immediately after electroporation, zygotes were transferred to pseudopregnant foster C57BL/6J females, and the pups, born about 3 weeks later were genotyped at the level of genomic DNA, extracted from the tail. Mice harboring the His6 tagged Defa30 protein were identified and crossed to C57BL/6J mice to start new transgenic families.

Supplemental Figure 3

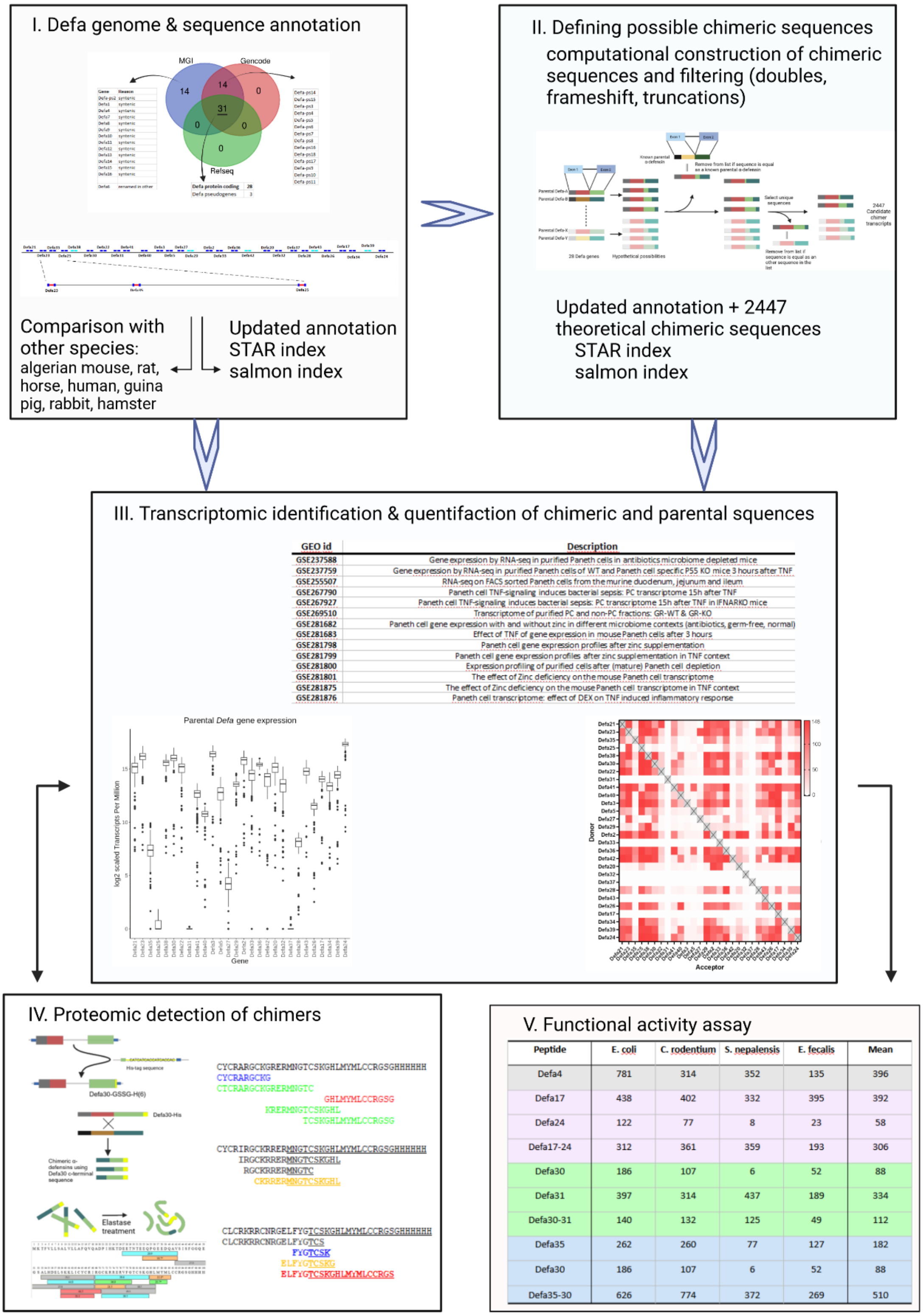

GEO id	Description
GSE237588	Gene expression by RNA-seq in purified Paneth cells in antibiotics microbiome depleted mice
GSE237759	Gene expression by RNA-seq in purified Paneth cells of WT and Paneth cell specific P55 KO mice 3 hours after TNF
GSE255507	RNA-seq on FACS sorted Paneth cells from the murine duodenum, jejunum and ileum
GSE267790	Paneth cell TNF-signaling induces bacterial sepsis: PC transcriptome 15h after TNF
GSE267927	Paneth cell TNF-signaling induces bacterial sepsis: PC transcriptome 15h after TNF in IFNARKO mice
GSE269510	Transcriptome of purified PC and non-PC fractions: GR-WT & GR-KO
GSE281682	Paneth cell gene expression with and without zinc in different microbiome contexts (antibiotics, germ-free, normal)
GSE281683	Effect of TNF of gene expression in mouse Paneth cells after 3 hours
GSE281798	Paneth cell gene expression profiles after zinc supplementation
GSE281799	Paneth cell gene expression profiles after zinc supplementation in TNF context
GSE281800	Expression profiling of purified cells after (mature) Paneth cell depletion
GSE281801	The effect of Zinc deficiency on the mouse Paneth cell transcriptome
GSE281875	The effect of Zinc deficiency on the mouse Paneth cell transcriptome in TNF context
GSE281876	Paneth cell transcriptome: effect of DEX on TNF induced inflammatory response

Peptide	E. coli	C. rodentium	S. nepalensis	E. fecalis	Mean
Defa4	781	314	352	135	396
Defa17	438	402	332	395	392
Defa24	122	77	8	23	58
Defa17-24	312	361	359	193	306
Defa30	186	107	6	52	88
Defa31	397	314	437	189	334
Defa30-31	140	132	125	49	112
Defa35	262	260	77	127	182
Defa30	186	107	6	52	88
Defa35-30	626	774	372	269	510

Supplemental Fig 3. Workflow of the study. We processed the genome annotations to find a core set of 28 coding defensin genes and used those to remap &quantify our RNA-seq datasets and to compare to other species. We made a set of potential chimeric defensin sequences and added those to the transcriptome set and used the updated set for detection & quantification of chimeric transcripts. In parallel, we created a transgenic mouse in which the *Defa30* gene was modified so that the DEFA30 protein would have a C-terminal His6 tail, in order to purify paternal DEFA30 protein as well as proteins derived from chimeric Defa genes, in which Defa30 acts as an acceptor parent. Finally, we used synthetic peptides to perform functional assays to show that chimers can have biological activity comparable to the classical defensins.

Supplemental Figure 4

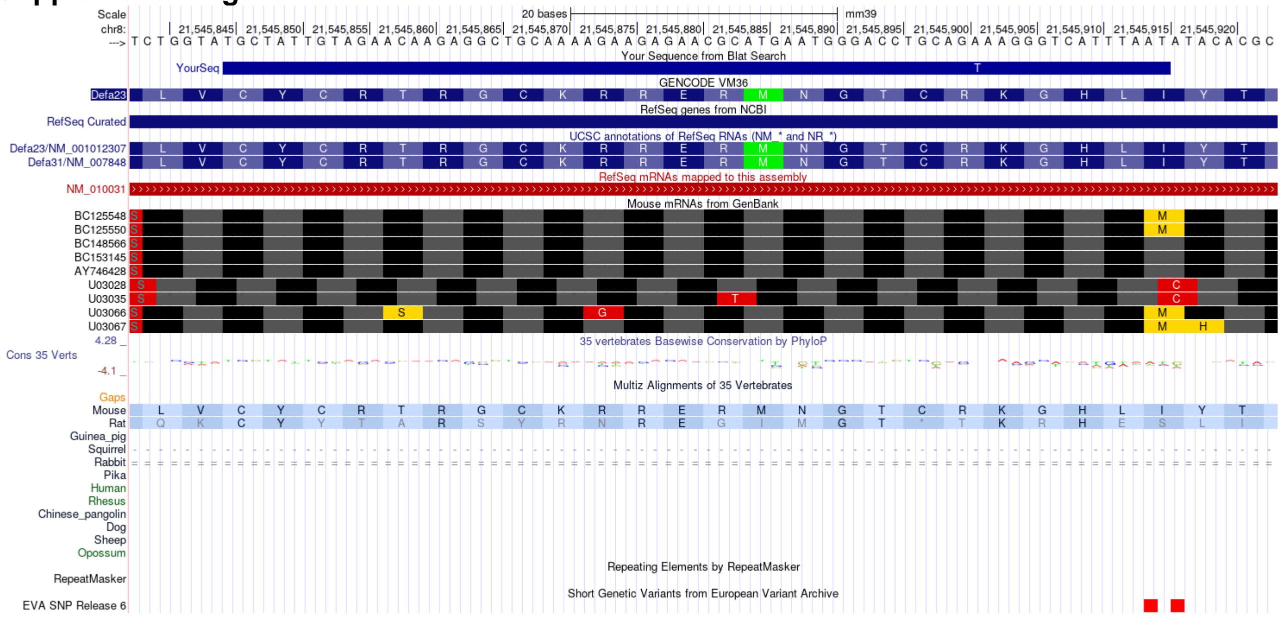

Supplemental Fig 4. Blat of against mm39 - Defa23: a blat of the read with sequence "ATTAAATGACCCT-TACTGCAGGTCCCATTCATGCGTTCTCTTCTTTTGCAGCCTCTTGTTCTACAATAGCA" mapping to Defa23 with one mismatch in at the end of the sequence.

Supplemental Figure 5

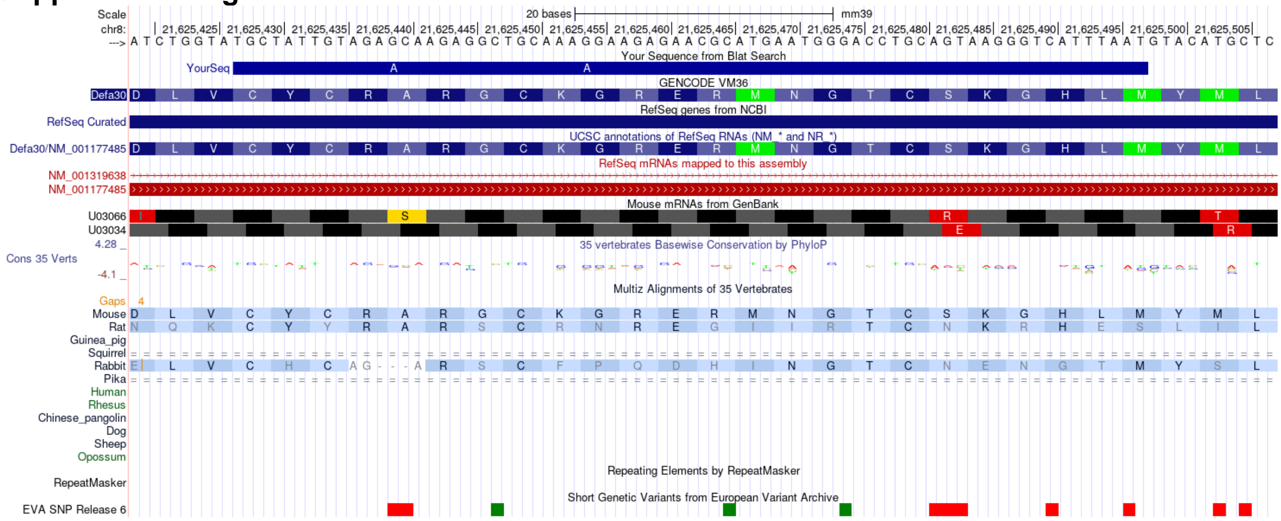

Supplemental Fig 5. Blat of against mm39 - Defa30: a blat of the read with sequence “ATTAAATGACCCT-TACTGCAGGTCCCATTCATGCGTTCTCTTCTTTTGCAGCCTCTTGTTCTACAATAGCA” mapping to Defa30 with two mismatches in the first half of the sequence.

Name Transcript	AVG TPM	SD TPM
Defa24	142928.28571	4380.12429
Defa3	73145.51429	2386.9493
Defa2	69726.41429	4411.06102
Defa23	68099.3	4383.16038
Defa20	55238.82857	4095.06726
Defa30	53390.04286	6998.02378
Defa21	51533.48571	3437.37052
Defa22	50708.8	3334.66966
Defa43	48345.38571	4252.4542
Defa36	46092.82857	6020.00678
Defa38	40751.52857	3729.8117
Defa33	33070.3	3923.13829
Defa39	21256.75714	2001.76207
Defa32	20591.22857	2249.14413
Defa17	15630.91429	887.85935
Defa29	15058.64286	1102.60534
Defa34	14299.09571	3241.82729
Defa42	10740.29286	1160.61308
Defa5	9566.14857	478.34157
Defa41	6521.94	818.98301
Defa26	2649.82714	324.15328
Defa40	1971.51	305.5357
Defa28	385.68029	41.31393
Defa35	171.17324	36.03371
Defa27	35.8387	10.37225
Defa25	0.38097	0.38224
Defa31	0	0
Defa37	0	0
Sum	851910.14463	

Supplemental Table 1. RNA-seq results of a typical experiment with 3 C57BL/6J mouse Paneth cell samples. The RNA-seq data were pooled and normalized to a total of 1 million reads (the actual amount of reads was around 25 million). Tab 1 shows that we detected 69.737 unique transcripts. They are listed from most to less abundant. In Tab 2 we display the Taggs Per Million (TPMs) of the *Defa* genes.

Gene transcripts	Median Fig 2C (log2 TPM)	Median Fig 3A (log2 TPM)
Defa21	15.2133	13.97258
Defa23	16.21241	15.07588
Defa35	7.44309	0
Defa25	0	0
Defa38	15.65395	14.6906
Defa30	16.01429	14.2922
Defa22	15.24835	7.83041
Defa31	0	0
Defa41	12.75634	0
Defa40	10.80691	7.6143
Defa3	16.43902	5.4592
Defa5	12.81905	0
Defa27	4.27896	0
Defa29	13.58081	11.87055
Defa2	15.87877	15.32284
Defa33	14.56545	13.4464
Defa36	15.40819	12.66386
Defa42	14.28817	12.39496
Defa20	15.22922	5.51276
Defa32	13.62101	0
Defa37	0	0
Defa28	8.28569	6.99844
Defa43	14.80376	14.27652
Defa26	11.6041	9.1782
Defa17	14.05222	0
Defa34	13.43103	0
Defa39	14.44368	13.57489
Defa24	17.3268	15.86268

Supplemental Table 2. The median values of the Defa gene expression levels as TPM as they are shown in Fig. 2C and in Fig. 3A.

	Defa21	Defa23	Defa35	Defa25	Defa38	Defa30	Defa22	Defa31	Defa41	Defa40	Defa3	Defa5	Defa27	Defa29	Defa2	Defa33	Defa36	Defa42	Defa20	Defa32	Defa37	Defa28	Defa43	Defa26	Defa17	Defa34	Defa39	Defa24
Defa21	NA	5.8968	2.32971	13.83005	7.6936	2.74177	11.45759	0	5.006	1.83568	8.30772	3.97596	0	6.32652	5.7997	5.85186	5.7542	6.32591	7.67591	4.32603	0	6.26353	5.41792	0.171	7.47022	2.45855	0.51525	3.26122
Defa23	8.88846	NA	3.68684	15.24025	8.49132	5.73369	4.28817	0	2.64149	3.1961	3.39914	4.12769	3.36841	8.53607	8.75471	7.45464	7.12055	4.20682	3.39864	3.26032	0	4.51354	3.73086	5.02209	4.86159	5.97674	2.73429	2.23071
Defa35	1.16989	1.87655	NA	6.85152	1.21029	3.60836	0	0	0	0	0	5.94786	0	3.24079	2.94129	4.7357	5.81176	0	0	0	0	2.14316	0	2.0565	2.51587	4.03511	1.67576	0
Defa25	0	3.52436	0	NA	0	0	0	0	0	0	0	0	3.59416	0	0	0	1.0298	0	0	0	0	0.27569	0	0	0.81796	1.75777	0	0
Defa38	3.18436	5.27524	0	15.16818	NA	4.53549	2.57244	0	2.19452	0.63703	5.56429	4.3259	1.34296	6.16956	4.26521	3.27386	4.29491	7.62375	5.06129	4.47411	0	0.60775	5.01423	1.4724	5.99412	4.3896	3.26124	4.85697
Defa30	9.5282	3.16436	6.83572	15.85388	10.03664	NA	4.02403	0	4.35703	6.45115	1.43028	3.23865	0	9.99939	9.49713	8.21154	5.14983	5.1512	3.3714	3.5662	0	8.09344	6.35345	7.24729	4.26922	1.98622	6.13485	6.66013
Defa22	13.91653	5.74369	2.02097	13.88594	8.1971	2.01642	NA	0	5.10034	1.32105	8.17542	3.75985	0	6.22188	5.9906	6.06041	5.2986	6.48121	6.69043	3.70155	0	5.61541	5.42237	1.27578	7.35622	3.10739	0.01576	2.35783
Defa31	0	0	0	0	0	0	0	NA	0.22429	0	0	0	0	0	0	0	0	0	0	0	0	0	0	0	0	0	0	0
Defa41	6.1033	4.87538	6.27291	11.23285	4.19797	3.27689	0	0	NA	0	0	9.02943	0	1.44053	5.68278	3.85013	13.2252	0	0	0	0	0.13927	2.3398	0.65011	5.79121	9.49423	1.13905	3.85885
Defa40	4.6226	1.11214	0	10.77032	3.66243	0	0	0	1.74485	NA	1.31501	0	0	3.97283	4.49364	2.75525	1.65411	0	0	0	0	0.0036	2.28365	8.55846	3.06983	0	1.01248	0.52478
Defa3	9.20898	8.39951	5.04796	15.97007	9.84378	5.97863	4.96483	0	4.14222	4.40532	NA	4.10768	0.45863	9.44413	9.18411	8.20351	7.45667	6.26655	4.03112	2.5465	0	7.31295	4.84841	5.61713	15.3437	5.46026	4.21167	2.42481
Defa5	4.72734	5.71185	0	12.88461	6.61308	6.48038	0.89583	0	3.27327	5.41662	4.64871	NA	1.16924	6.92905	6.63112	7.98407	6.92701	0.79411	1.59423	1.36424	0	6.88788	4.85417	6.75126	7.4676	8.62186	4.34672	8.56177
Defa27	0	3.19908	0	0	0	0	0	0	0	0	4.28485	0	NA	0	0	0	3.38542	0	0	0	0	0	0	0	3.02849	4.54782	0	0
Defa29	2.51387	3.78074	0	10.73541	7.5722	3.022	2.93923	0	1.95708	3.2088	2.81042	0	0	NA	0.68057	0	1.86094	4.76437	1.78385	1.03685	0	2.95322	5.85962	3.00086	4.33371	2.59952	6.13506	3.17432
Defa2	9.16371	5.63336	2.54161	15.02356	9.1269	1.7597	5.24776	0	6.72814	0.29481	6.59997	4.79029	0	6.07451	NA	0	4.64977	5.87941	13.91196	10.94252	0.22429	0	7.61177	0	6.86299	3.48081	3.84435	5.39687
Defa33	0	0	0	0	0	0	0	0	0	0	0	0	0	0	0	NA	0	0	0	0	0	0	0	0	0	0	0	0
Defa36	7.87757	8.51314	6.86082	15.07878	7.63397	8.88557	2.07425	0	4.41171	4.38183	6.3765	7.82723	1.37679	9.56802	8.43356	7.2866	NA	1.08455	2.54964	1.56226	0	6.81383	6.13172	4.5654	8.75283	8.85612	6.27452	7.03152
Defa42	7.42203	8.48819	1.01254	13.57239	10.23129	8.0207	6.68173	0	0	3.27498	8.05473	5.31916	0	8.71002	8.64329	7.68752	6.8501	NA	8.66315	5.79409	0	3.07211	0	3.90261	8.90222	7.10772	7.68703	9.0605
Defa20	3.05245	8.68381	5.41134	10.44978	9.51624	3.77979	4.2948	0	7.86309	0	6.32188	3.56592	0	6.4884	10.89111	9.62366	5.49529	5.58675	NA	0	0	10.04157	4.81701	1.96878	10.39245	3.34829	4.47144	5.23699
Defa32	0	0	0	0	0	0	0	0	0	0	0	0	0	0	0	0	0	0	0	NA	0	0	0	0	0	0	0	0
Defa37	0	0	0	0	0	0	0	0	0	0	0	0	0	0	0	0	0	0	0	0	NA	0	0	0	0	0	0	0
Defa28	0.20939	0.99909	0	7.29456	0.27366	0	0	0	0	0	0	2.61398	0	0	0.1078	0	3.51604	0.43262	0	0	0	NA	1.47408	0.56416	1.83384	2.01555	0	0
Defa43	0	0	0	0	0	0	0	0	0	0	0	0	0	0	0	0	0	0	0	0	0	0	NA	0	0	0	0	0
Defa26	8.06356	7.55195	0	11.30255	5.73944	6.57206	0	0	1.14743	7.85969	4.57037	0	0	5.21628	8.52787	6.25822	7.57566	4.08596	1.39985	0	0	0.83633	6.22382	NA	8.86992	3.48209	5.45529	7.97259
Defa17	0	0	0	0	0.5135	0	0	0	0	0	0	0	0	0	0	0	0	0	0	0	0	0	0	0	NA	0	0	0
Defa34	7.16808	6.69405	0	13.58315	6.07531	8.04543	0.3332	0	1.11896	5.14864	5.62993	10.51078	0	7.43612	7.17872	7.26994	7.33387	0	0.93579	0.92591	0	5.44627	4.05044	6.1011	9.00285	NA	4.58362	7.11138
Defa39	4.01147	6.21124	0	12.68194	9.51601	5.35682	7.27026	0	2.99997	1.49727	4.79727	6.66482	0	10.6637	5.02989	4.56554	4.84377	6.14058	7.80215	6.22542	0	1.65319	8.80578	2.36614	7.07684	3.81932	NA	6.23398
Defa24	9.77048	3.03882	6.05664	16.61089	10.89365	12.83519	5.37336	0	4.33744	6.49753	2.61237	3.82018	0	10.83067	9.38328	7.16724	3.79082	6.58339	4.72208	4.26476	0	6.33689	6.39782	7.26999	2.50882	4.04195	5.3264	NA

Supplemental Table 3. The actual (not log2) TPM plotted in the heat map of Fig. 3D. Over 148 bulk RNA-seq experiments, in which all reads in each experiment are normalized to 1 million, the observed chimeric mRNAs remain low in expression.
